# Assessment of a novel BLOOMY score for predicting mortality in hospitalised adults with bloodstream infection

**DOI:** 10.1007/s15010-024-02254-5

**Published:** 2024-04-23

**Authors:** Johanna Tietäväinen, Tapio Seiskari, Janne Aittoniemi, Heini Huhtala, Jukka Mustonen, Reetta Huttunen, Jaana Syrjänen, Juha Rannikko

**Affiliations:** 1 Department of Internal Medicine, Tampere University Hospital, P.O. Box 2000, 33521 Tampere, Finland; 2 Faculty of Medicine and Health Technology, Tampere University, Arvo Ylpön Katu 34, 33520 Tampere, Finland; 3 Department of Clinical Microbiology, Fimlab Laboratories Ltd, P.O. Box 66, 33013 Tampere, Finland; 4 Faculty of Social Sciences, Tampere University, Arvo Ylpön Katu 34, 33520 Tampere, Finland

**Keywords:** Sepsis, Bacteraemia, Score, Machine learning

## Abstract

**Purpose:**

A German multicentre study BLOOMY was the first to use machine learning approach to develop mortality prediction scores for bloodstream infection (BSI) patients, but the scores have not been assessed in other cohorts. Our aim was to assess how the BLOOMY 14-day and 6-month scores estimate mortality in our cohort of 497 cases with BSI.

**Methods:**

Clinical data, laboratory data, and patient outcome were gathered retrospectively from patient records. The scores were calculated as presented in the BLOOMY study with the exception in the day of the evaluation.

**Results:**

In our cohort, BLOOMY 14-day score estimated death by day 14 with an area under curve (AUC) of 0.87 (95% Confidence Interval 0.80–0.94). Using ≥ 6 points as a cutoff, sensitivity was 68.8%, specificity 88.1%, positive predictive value (PPV) 39.3%, and negative predictive value (NPV) 96.2%. These results were similar in the original BLOOMY cohort and outweighed both quick Sepsis-Related Organ Failure Assessment (AUC 0.76) and Pitt Bacteraemia Score (AUC 0.79) in our cohort. BLOOMY 6-month score to estimate 6-month mortality had an AUC of 0.79 (0.73–0.85). Using ≥ 6 points as a cutoff, sensitivity was 98.3%, specificity 10.7%, PPV 25.7%, and NPV 95.2%. AUCs of 6-month score to estimate 1-year and 5-year mortality were 0.80 (0.74–0.85) and 0.77 (0.73–0.82), respectively.

**Conclusion:**

The BLOOMY 14-day and 6-month scores performed well in the estimations of mortality in our cohort and exceeded some established scores, but their adoption in clinical work remains to be seen.

**Supplementary Information:**

The online version contains supplementary material available at 10.1007/s15010-024-02254-5.

## Introduction

Various scores have been developed for emergency department and hospitalised patients to determine the degree of illness or to predict an endpoint such as mortality. An example of a widely used score for all patients is National Early Warning Score 2 [1]. The vital signs or other parameters needed for the score are inserted in an electronic patient record and the record automatically shows you, for example, the trend of the score. Another example is Sequential Organ Failure Assessment (SOFA) score [2], commonly used for all intensive care unit (ICU) patients to predict mortality. The newcomers, BLOOMY 14-day and 6-month prediction scores [3], are developed for bloodstream infection (BSI) patients, they need to be calculated electronically, and they have not been yet studied in other cohorts than in the original BLOOMY study. Before roll-out, more studies are needed to test their reproducibility and validity [4–7].

The BLOOMY study is a multicentre cohort study at six German tertiary care university hospitals. It was the first study to develop a prediction score for patients with infection by use of machine-learning approach. The 14-day and 6-month mortality model predictors included, for example, age, body-mass index, platelet counts and C-reactive protein concentrations (see Tables 1 and 2). The total possible scores for the BLOOMY 14-day and 6-month score were 20 and 26, respectively. In prediction of mortality, sensitivity increased with higher scores but specificity decreased; the study group used mostly 6 or 7 points as cutoff. The researchers had a separate validation cohort to calculate predictive accuracy. The aim of our study was to assess how the score estimates mortality in our cohort of 497 Finnish BSI patients, i.e., to externally assess the sensitivity, specificity, and other predictive values. We also studied how quick SOFA (qSOFA) is comparable to BLOOMY 14-day score and tested how BLOOMY 6-month score predicts 1-year and 5-year mortality.

**Table 1:** Points allocations for BLOOMY 14-day and quick BLOOMY 14-day scores by variable assessed, as presented by Tacconelli et al. [3]

	Points allocated
BLOOMY 14-day score	
Age, years	
≥ 80 and not on mechanical ventilation	1
≥ 80 and on mechanical ventilation	3
Body mass index ≤ 20 kg/m^2^	1
Pathogen: Pseudomonas aeruginosa, Enterococcus faecium, or methicillin-resistant Staphylococcus aureus	1
Estimated glomerular filtration rate ≤ 30 mL/min per 1.73 m^2^	2
Late hospital acquisition (> 14 days length of hospital stay)	2
Metastatic malignancy	1
Hypotension	1
Mental status	
Disorientation	2
Stupor or comatose	3
Leukocyte count ≥ 30,000/μL	2
Thrombocyte count ≤ 50,000/μL	2
C-reactive protein ≥ 20 mg/dL	2
Quick BLOOMY 14-day score	
Age, years	
40–79	2
≥ 80	3
Hypotension	2
Mental status	
Disorientation	2
Stupor	3
Comatose	4

**Table 2:** Points allocations for BLOOMY 6-month and quick BLOOMY 6-month scores by variable assessed, as presented by Tacconelli et al. [3]

	Points allocated
BLOOMY 6-month score	
Age ≥ 80 years	2
Body mass index ≤ 20 kg/m^2^	2
Pathogen: Staphylococcus aureus, vancomycin-resistant Enterococcus faecium, or multidrug-resistant Gram-negative bacteria	1
Estimated glomerular filtration rate ≤ 60 mL/min per 1.73 m^2^	1
Hospital-acquired infection	1
Malignancy	
No metastasis and no chemotherapy	2
Having chemotherapy	5
Metastasis	4
Complications	
Septic shock, liver failure, lung failure, or kidney failure	1
Secondary dissemination, disseminated intravascular coagulopathy, or thrombosis or decubitus	2
Focus of infection	
Abdominal	1
Respiratory	2
Leukocytes, per μL	
8000 to < 13,000	1
≥ 13,000	3
Thrombocytes, per μL	
≤ 50,000	3
> 50,000 to 150,000	2
C-reactive protein, mg/dL	
5 to < 20	2
≥ 20	4
Quick BLOOMY 6-month score	
Age ≥ 80 years	1
Malignancy with metastasis	2
Leukocytes, per μL	
8000 to < 13,000	1
≥ 13,000	2
C-reactive protein, mg/dL	
5 to < 20	1
≥ 20	2
Thrombocytes, per μL	
> 50,000 to 150,000	1
≤ 50,000	2

## Materials and methods

### Bacteraemia patient cohort

Tampere University Hospital is a tertiary hospital with a catchment population of ca. 525 000 inhabitants in the Pirkanmaa County, Finland. The study cohort included consecutive blood culture-positive patients in the Tampere University Hospital ED between March 1, 2012 and February 28, 2014. The cases in which positive blood culture were determined as contamination were excluded. The clinical data of the patients were collected retrospectively from patient records. A criterion for the qSOFA score was calculated based on Sepsis‐3 definitions [8]. The Pitt Bacteraemia Score was calculated as presented by Korvick et al. [9]. The site of infection was determined retrospectively. A more detailed description of the patient cohort is available in our previous publications [10–12]. The study was approved by the Ethics Committee of Tampere University Hospital, Finland (permit# R11099). The need for informed consent was waived as routine patient care was not modified.

### Variables

The BLOOMY 14-day and 6-month scores were calculated as described in the original article apart from two modifications due to practical reasons. On BLOOMY 14-day scores, day 0 variables were used instead of day 3 variables. This has an effect on points allocated to hypotension, mental status, and laboratory values (see Table 1), but not on the permanent patient data such as malignancies and medication. BLOOMY 6-month score uses laboratory variables minimum of six days after the last day of antibiotic treatment (Table 2). Instead, day 0 laboratory parameters were used. Again, permanent patient date was collected as in original article. In dichotomous variables, only cases with missing data that would affect the result were excluded. In continuous variables all cases with missing data were excluded. Patients with multiple admissions (*n* = 12) were handled as they would be separate cases.

### Statistical analyses

SPSS version 28.0 software (IBM Corp., NY, USA) was used for statistical analyses. AUC analysis was used to assess the scores’ discriminative value and specificity, sensitivity, negative predictive value (NPV), and positive predictive value (PPV) was used to assess the predictive accuracy. A *P*‐value of < 0.05 was considered significant. To assess the calibration of the models, the calibration belt was used [7, 13].

## Results

A total of 497 cases (484 patients) with positive blood culture were included. Median age was 68 (range 16–95 years), 53% were male, and Gram-positive and Gram-negative bacteraemia were equally common. Other characteristics, the severity of the disease, and causative organism are shown in Table 3. Ninety-nine cases were missing weight and 3 cases day of admission C-reactive protein, no other missing data for the BLOOMY score calculations. We found no need to recalibrate the scores (see supplement report).

**Table 3:** Patient characteristics, severity of the disease and microbiological data of the study population

	n (%)
Demographic	
Cases/patients	497/484
Patients admitted twice/three times	11/1
Median age, years	68 (range 16–95)
Sex (male)	262 (53)
Chronic medical condition	
Coronary artery disease, chronic vascular disease, or chronic heart failure	176 (35)
Diabetes mellitus, any type	139 (28)
Social or medical problems of alcohol abuse	52 (11)
Solid tumour with metastasis	57 (12)
Haematological malignancy	45 (9)
No underlying diseases	71 (14)
Site of infection	
Urinary	134 (27)
Intra-abdominal	84 (17)
Skin, soft tissue and bones	72 (14)
Pulmonary	54 (11)
Others	35 (7)
Unknown	118 (24)
Causative organism	
Gram-positive	225 (45)
*Staphylococcus aureus*	74 (15)
Methicillin-resistant *Staphylococcus aureus*	3 (0.6)
Gram-negative	223 (45)
*E. coli*	159 (32)
Extended-spectrum beta-lactamase Enterobacterales	9 (2)
Carbapenemase-producing Enterobacterales	0 (0)
Others	
Anaerobes	15 (3)
Fungi	1 (0.2)
Polymicrobial	33 (7)
Severity of the bacteraemia	
qSOFA score ≥ 2^a^	136 (29)
Septic shock	37 (7)
Pitt Bacteraemia Score^b^	
0–1	306 (62)
2–3	123 (25)
≥ 4	61 (12)
Day of case fatality	
Days 0 to 14	56 (11)
Days 0 to 180	121 (24)

^a^Data available on 473 cases

^b^Data available on 490 cases

We calculated the predictive value of BLOOMY 14-day score to estimate death by day 14. AUC was 0.87 (95% Confidence Interval 0.80–0.94). Using ≥ 6 points as a cutoff, sensitivity was 68.8% and specificity 88.1%. With ≥ 7 points, sensitivity was 42.9%, specificity 94.9%, PPV 48.8%, and NPV 93.5%. In our cohort, Pitt Bacteraemia Score had an AUC of 0.79 (0.72–0.86) to estimate 14-day mortality. For the same estimation, ≥ 2 qSOFA points had an AUC of 0.76 (0.69–0.83).

Using ≥ 6 points as a cutoff, BLOOMY 6-month score estimated 6-month mortality with a sensitivity of 98.3% and a specificity of 10.7%. Increasing cutoff to ≥ 7 points decreased sensitivity to 93.2% and increased specificity to 21.0% (data on 483 cases). BLOOMY 6-month score had an AUC of 0.79 (0.73–0.85). AUCs of 6-month score to estimate 1-year and 5-year mortality were 0.80 (0.74–0.85) and 0.77 (0.73–0.82), respectively; see Table 4 for other predictive values for both BLOOMY 14-day and 6-month score.

**Table 4:** The predictive values of BLOOMY 14-day/6-month score in the original validation cohort and BLOOMY 14-day/6-month and qSOFA score ≥ 2 in our cohort

	Cases	Score positive in deceased/all deceased	Sensitivity (%)	Specificity (%)	Positive predictive value (%)	Negative predictive value (%)	Odds ratio with 95%CI	AUC with 95%CI^a^
Death by day 14								
Original validation cohort BLOOMY 14-day score ≥ 6	898	65/106	61.3	86.4	37.6	94.4	NA	0.86 (0.82–0.89)
Our cohort BLOOMY 14-day score ≥ 6	476^b^	33/48	68.8	88.1	39.3	96.2	16.3 (8.3–32.0)	0.87 (0.80–0.94)^c^
Our cohort Pitt Bacteraemia Score ≥ 2	490	41/54	75.9	67.2	22.3	95.8	6.5 (3.4–12.4)	0.79 (0.72–0.86)
Our cohort qSOFA score ≥ 2	494	42/56	75.0	77.5	30.9	95.9	10.3 (5.4–19.7)	0.76 (0.69–0.83)
Death by month 6								
Original validation cohort BLOOMY 6-month score ≥ 6	575	NA	79.0	52.8	35.7	88.4	NA	0.74 (0.70–0.79)
Our cohort BLOOMY 6-month score ≥ 6^d^	490^e^	115/117	98.3	10.7	25.7	95.2	6.9 (1.6–29.0)	0.79 (0.73–0.85)
Our cohort Pitt Bacteraemia Score ≥ 2	490	68/118	57.6	68.8	37.0	83.7	3.0 (2.0–4.6)	0.68 (0.62–0.74)

Abbreviation: *NA* not available

^a^Area under curves (AUCs) are calculated as continuous variable except qSOFA

^b^Cases with 5 points and missing weight excluded (21 cases)

^c^Data on 397 cases (99 cases with missing weight excluded)

^d^See methods for the difference in calculations of BLOOMY 6-month score

^e^Cases with 4 or 5 points and missing weight excluded (7 cases)

The quick BLOOMY 14-day score included three parameters: age, mental status, and hypotension. The predictive value (AUC) of quick BLOOMY 14-day score to estimate 14-day mortality was 0.81 (0.75–0.87). Quick BLOOMY score of ≥ 6 points predicted 19 of 55 deaths at 14 days with a sensitivity of 34.6%, a specificity of 95.5%, a PPV of 48.7%, and an NPV of 92.1%.

## Discussion

We calculated the predictive value of BLOOMY 14-day score to estimate death by day 14. In our study AUC was 0.87, almost the same as in the original BLOOMY cohort (0.86). Using 6 points or more as a cutoff, our sensitivity to predict day 14 mortality was 68.8% (original 61.3%) and specificity 88.1% (original 86.4%). Quick BLOOMY 14-day score AUC was 0.81 in our cohort vs. 0·83 in the BLOOMY cohort. The AUC of Pitt Bacteraemia Score to estimate 14-day mortality was 0.79, almost identical than in the original cohort (0.78). The AUC of qSOFA was 0.76 in our study. In the BLOOMY study qSOFA was not calculated.

The predictive values of BLOOMY 6-month score to estimate 6-month mortality in our cohort were different from the original BLOOMY cohort. We calculated the score by using day 0 laboratory values instead of, as in the BLOOMY cohort, the last day of antibiotic treatment plus minimum of six days. This affected the specificity of the 6-point cutoff; it was much lower in our cohort (10.7%) than in the BLOOMY cohort (52.8%). However, AUC was still higher than in the original cohort, 0.79 vs. 0.74, respectively.

The BLOOMY study compared the new scores to Pitt Bacteraemia Score, Gram-Negative Bloodstream Infection Risk Mortality Score [14], and, indirectly, to results from a qSOFA meta-analysis [15]. They found the predictive values of BLOOMY 14-day and 6-month scores to be better than in these older scores. Combined with our results, it appears that the machine learning approach has developed a score that yields better 14-day and 6-month mortality estimations than these established scores.

BLOOMY 14-day and 6-month prediction scores are developed for BSI patients, and they need to be calculated electronically. One prediction score developed for patients with infection, e.g. qSOFA, is, in contrast to all BLOOMY scores, easily calculated bedside and therefore gained popularity. According to our experience, other prediction scores developed only for patients with infection have not been widely used in clinical settings. Pitt Bacteraemia Score would need either electronic record-based, web-based, or app-based calculation to obtain the results, but these calculations are rarely performed. The same might happen with these newcomers.

Our study had some limitations. We were able to calculate all BLOOMY scores retrospectively, but some values were missing. Ninety-nine cases were missing weight which is used in BLOOMY 14-day and 6-month score (BMI ≤ 20 kg/m^2^ equals 1 point). We decided not to reduce the total number to those with weight as the missing values affected less on the dichotomous variables (see Table 4). We had > 99% of other values. Other limitation is the difference in the days of evaluation in the original cohort and in our cohort. The original cohort used day 3 as an evaluation point in BLOOMY 14-day score and the last day of antibiotic treatment plus minimum of 6 days in BLOOMY 6-month score. In the present study, day 0 was used as an evaluation point in both BLOOMY 14-day and in the laboratory results of BLOOMY 6-month score. The difference in BLOOMY 14-day evaluation day can also been seen as an advantage of the present study, but the difference in BLOOMY 6-month might have been the reason for the differences in the sensitivity and specificity in the original and this study as the laboratory results add up to maximum of 10 points out of 26 total points. On the other hand, in clinical work, laboratory tests are often not routinely taken 6 days after antibiotic treatment.

In conclusion, the BLOOMY 14-day and BLOOMY 6-month scores performed well in our cohort. However, it is more likely that any new score is adopted in clinical routine if it is for general patient population and not a score for cases with a certain laboratory result (i.e. positive blood culture). It is possible that these new scores will remain mostly in research purposes.

### Supplementary Information

Below is the link to the electronic supplementary material.

Supplementary file1 (DOCX 77 KB)

## Data Availability

Data available on request.
