# Supplementary material for: Assessment of a novel BLOOMY score for predicting mortality in hospitalised adults with bloodstream infection
Source: Infection. 2024 Apr 23;52(4):1511–7. doi: 10.1007/s15010-024-02254-5 (PMC11289069; doi:10.1007/s15010-024-02254-5)
Supplement: Supplementary file 1 — Supplementary file1 (DOCX 77 KB) [file 15010_2024_2254_MOESM1_ESM.docx]

**Supplemental calibration report**

To assess the calibration of the models, the calibration belt was used. That is a curve that represents the association between predicted and observed values and their confidence intervals (80% and 95%) throughout all the risk ranges. Cases were randomly divided into two groups.

The figures 1 and 3 (development sample) suggests that the predictions of the models do not significantly deviate from the observed. The models’ internal calibrations are acceptable.

The figures (2 and 4) suggest that the fitted models are well calibrated in the validation sample.

Based on these results we found no need to recalibrate the scores.

Stata 18.0 (College Station, Texas 77845 USA)

1. **14-days score internal**

1. **14-days score external**

1. **6-month score internal**

1. **6-month score external**
